# Tailor-made notched music training–induced residual inhibition in subjective tinnitus: resting-state EEG spectral power ratio evidence

**DOI:** 10.3389/fnins.2026.1732336

**Published:** 2026-02-11

**Authors:** Jiaqing Hu, Ziyue Zhang, Wei Wang

**Affiliations:** 1 Department of Otorhinolaryngology Head and Neck Surgery, Tianjin First Central Hospital, Tianjin, China; 2 Institute of Otolaryngology of Tianjin, Tianjin, China; 3 Key Laboratory of Auditory Speech and Balance Medicine, Tianjin, China; 4 Key Medical Discipline of Tianjin (Otolaryngology), Tianjin, China; 5 Quality Control Centre of Otolaryngology, Tianjin, China; 6 First Central Clinical College, Tianjin Medical University, Tianjin, China

**Keywords:** EEG, residual inhibition, spectral power ratio, tailor-made notched music training (TMNMT), tinnitus

## Abstract

**Objective:**

Residual inhibition (RI) is clinically useful but lacks objective markers. Tailor-made notched music training (TMNMT) is a non-invasive paradigm that may elicit RI. We evaluated whether resting-state EEG spectral power ratios (SPR) provide a convergent electrophysiological signature of TMNMT-induced RI.

**Methods:**

We retrospectively analyzed 29 adults with chronic subjective tinnitus who underwent a single 10-min TMNMT session. Resting-state EEG was recorded immediately before (pre-stim), during (during-stim), and after stimulation (post-stim). Power was estimated by Welch’s method and summarized as band-limited SPR for delta (1–4 Hz), theta (4–8 Hz), alpha (8–13 Hz), and beta (13–30 Hz) relative to broadband power (1–30 Hz). Behavioral outcomes included visual analogue scales (VAS) for loudness/annoyance and RI intensity and duration. Repeated-measures non-parametric tests with false-discovery-rate (FDR) correction were the primary analyses; effect sizes were reported where applicable.

**Results:**

RI was observed in 20 of 29 patients (69%) following the routine TMNMT exposure. Across the cohort, VAS loudness/annoyance decreased within-session. SPR showed a frequency-specific shift characterized by increases in delta and theta power ratios and a reduction in alpha, with a non-significant downward trend in beta. Exploratory analyses suggested that higher baseline tinnitus severity (THI) was associated with greater VAS improvement and higher likelihood of RI positivity.

**Conclusion:**

The present retrospective analysis characterized behavioral and EEG spectral patterns observed during routine TMNMT exposure. We observed that TMNMT produced measurable tinnitus relief accompanied by frequency-specific SPR shifts. These findings collectively offer preliminary observational evidence and practical rationale for the potential clinical utility of TMNMT.

## 1 Introduction

Tinnitus refers to the perception of sound in the absence of an external acoustic stimulus (Cima et al., 2019) and affects approximately 10–15% of the adult population worldwide (Heller, 2003)^.^ While many cases are mild, some individuals suffer from debilitating symptoms such as irritability, insomnia, anxiety, depression, and even suicidal ideation, making tinnitus a serious global health concern (Tunkel et al., 2014). Although the exact pathophysiology of chronic tinnitus remains unclear (Cima et al., 2019), the prevailing explanation is the global brain network model (Schlee et al., 2011; Schlee et al., 2008), which posits two complementary mechanisms: (1) Reduced peripheral auditory input disrupts the balance of excitation and inhibition in the central auditory system, leading to increased spontaneous neural firing (Lanting et al., 2009; Møller, 2007)^,^growing evidence from animal (Dong et al., 2010) and human (Schlee et al., 2011; Schlee et al., 2008) studies links tinnitus to heightened excitability of auditory cortical regions. (2) Tinnitus is also associated with a non-auditory brain network, including regions involved in cognition, emotion, and memory (De Ridder et al., 2015; De Ridder et al., 2006; Golm et al., 2013; Vanneste et al., 2010)^.^

One important phenomenon observed in tinnitus research is Residual Inhibition (RI) — the temporary reduction in tinnitus loudness following acoustic stimulation (Spaulding, 1903; Feldmann, 1971). Over 75% of tinnitus patients experience some degree of RI (Roberts et al., 2006; Vernon and Meikle, 2003)^,^ with durations typically ranging from 5 to 30 s (Roberts et al., 2008). RI can be induced by various stimuli, including pure tones (Terry et al., 1983), broadband noise (Vernon and Meikle, 2003), narrow-band noise (Roberts et al., 2008)^,^ and amplitude-modulated sounds (Reavis et al., 2012; Tyler et al., 2014). The effectiveness depends on factors like stimulus intensity and frequency specificity, with stimuli matching the tinnitus frequency often yielding more effective suppression (Roberts et al., 2008; Roberts et al., 2006). RI is hypothesized to involve temporary suppression of abnormal auditory activity, particularly in deafferented regions caused by hearing loss (Roberts et al., 2008; Galazyuk et al., 2017; Galazyuk et al., 2019; Hu et al., 2021; Sedley et al., 2015). EEG and neuroimaging studies suggest that RI correlates with changes in alpha and delta bands (Sedley et al., 2015). RI holds significant value for understanding the mechanisms underlying tinnitus and shows considerable potential in clinical application, particularly as a prognostic indicator of individual patients’ responses to therapeutic acoustic stimulation (Roberts, 2007; Hu et al., 2021).

Among non-invasive therapies, sound therapy is strongly recommended by the American Academy of Otolaryngology–Head and Neck Surgery Foundation’s clinical practice guidelines for bothersome and persistent tinnitus (Tunkel et al., 2014). One promising approach is Tailor-Made Notched Music Training (TMNMT), which uses customized music with a frequency notch centered on the patient’s tinnitus frequency (Lugli et al., 2009; Okamoto et al., 2010). This induces lateral inhibition from neighboring frequencies and promotes neural plasticity in the auditory cortex (Stein et al., 2015; Pantev et al., 2012; Pantev et al., 2014). TMNMT has demonstrated significant efficacy: it improves self-assessment scores (e.g., Tinnitus Handicap Inventory, Visual Analogue Scale) (Diao et al., 2020) and reduces the loudness of tinnitus (Pantev et al., 2004; Wang et al., 2018). Recent studies (Zhu and Gong, 2023; Zhu and Gong, 2025) also report enhanced inhibitory function and longer-lasting effects of TMNMT.

A large body of electrophysiological research has identified abnormal oscillatory activity as a hallmark of tinnitus. MEG and EEG studies have consistently shown enhanced delta/theta activity and reduced alpha power in tinnitus patients relative to normal-hearing controls, reflecting altered inhibitory–excitatory balance and patterns consistent with thalamocortical dysrhythmia (Weisz et al., 2007; Weisz et al., 2005). These alterations have been replicated across several electrophysiological studies, including EEG evidence of reduced alpha and increased slow-wave synchronization in tinnitus cohorts (Moazami-Goudarzi et al., 2010), and reviews summarizing widespread abnormalities in low- and mid-frequency rhythms (Adjamian et al., 2012). Although some reports have identified divergent alpha findings, such variability is generally attributed to differences in tinnitus phenotypes, recording parameters, or analytic approaches. Oscillatory activity has also been shown to change following auditory interventions, including sound-based therapies. Studies have reported modulation of delta, theta, and alpha rhythms during or following tinnitus suppression, suggesting dynamic adjustment of cortical synchrony in relation to perceptual changes (Roberts et al., 2008; Sedley et al., 2015). Within this context, tailor-made notched music training (TMNMT) has demonstrated the capacity to reduce tinnitus loudness and alter auditory cortical responses via lateral inhibition and experience-dependent plasticity (Okamoto et al., 2010; Pantev et al., 2012). However, short-term oscillatory dynamics during TMNMT itself have been minimally explored, and the relationship between these neural changes and behavioral RI remains unclear.

Addressing this knowledge gap, the present retrospective study uses EEG-derived Spectral Power Ratio (SPR) to characterize oscillatory modulation during TMNMT and to examine its association with behavioral RI. By identifying frequency-specific neural signatures linked to immediate tinnitus suppression, this work aims to provide insight into the short-term neural mechanisms underlying sound-based tinnitus modulation and to inform individualized therapeutic strategies.

## 2 Materials and methods

### 2.1 Participants

This study was conducted at Tianjin First Central Hospital, Tianjin, and included 29 patients with tinnitus (15 males and 14 females), aged between 25 and 75 years. All participants underwent audiometry and otoscopy at enrolment by an otolaryngologist, to ensure data accuracy and reliability. Pure-tone audiometry was performed for all participants across the full clinical frequency range of 125 Hz to 8 kHz. For descriptive purposes, the pure-tone average (PTA) was calculated using thresholds at 0.5, 1, 2, and 4 kHz, following standard clinical convention. Tinnitus pitch matching was conducted independently up to 8.5 kHz, ensuring accurate determination of individualized notch-center frequencies even for patients with high-frequency tinnitus. Normal hearing was defined as a PTA below 20 dB HL, while mild hearing loss was defined as a PTA between 21 dB HL and 40 dB HL. Patients with severe hearing loss were excluded to ensure adequate perception of TMNMT. Among the 29 participants, 12 had normal hearing and 17 had mild hearing loss. Exclusion criteria were as follows: (1) history of substance abuse, including psychiatric medications or alcohol; (2) history of psychiatric or neurological disorders; (3) history of significant head trauma; (4) pulsatile tinnitus; (5) diagnosis of Meniere’s disease, external and middle ear lesions, and posterior cochlear lesions; (6) severe hearing loss. Additionally, Tinnitus Handicap Inventory (THI) and Visual Analogue Scale (VAS, including Loudness and Annoyance) were employed to evaluate the impact of tinnitus on participants’ daily lives. The study protocol was approved by the Tianjin First Central Hospital’s ethics committee (2020N114KY). Patients provided written permission for the use of their anonymized clinical data for research purposes, and no study-specific procedures or prospective enrollment were performed.

### 2.2 Tinnitus pitch and loudness matching tests

Tinnitus pitch and loudness matching were performed using a portable tinnitus treatment device. For pitch matching, 188 pure-tone stimuli covering the frequency range of 125 Hz to 8.5 kHz were presented in 1/30-octave steps, as described previously (Zhu and Gong, 2023). Participants listened to a series of tones and compared each with their perceived tinnitus until a tone that best matched their tinnitus pitch was identified. Once the tinnitus pitch was determined, loudness matching was conducted using the same device. The sound level was adjusted in 1 dB sound pressure level (SPL) increments until participants indicated that the loudness matched the perceived intensity of their tinnitus (Kim et al., 2016)^.^ This stepwise procedure allowed for fine adjustment to closely match each participant’s subjective tinnitus loudness. For bilateral and central tinnitus, tinnitus pitch and loudness were estimated separately for each ear.

### 2.3 Experimental procedure

This study was based on clinical data obtained during routine tinnitus assessment. As part of the standardized evaluation protocol, patients underwent EEG recording in a single session that consisted of three sequential phases. First, a 3-min resting-state EEG was recorded with eyes closed, followed by a 2-min eyes-open rest period. Subsequently, a 10-min tailor-made notched music stimulation (TMNMT) was presented at an individually adjusted level approximately 10 dB above the matched tinnitus loudness. For each patient, the audio stimulus was digitally notched at the tinnitus center frequency to enhance lateral inhibition at the notch edges. To improve stimulation efficiency in higher frequency bands, the spectral envelope was flattened by reallocating energy from lower to higher frequencies as described by Teismann et al. (2011). The TMNMT material consisted of naturalistic environmental sounds (e.g., rain, streams, waves), which are typically perceived as relaxing (Strauss et al., 2017). Audio was delivered binaurally via insert earphones. During TMNMT, EEG was recorded continuously with eyes closed (duration: 10 min).

After TMNMT, a further 5-min eyes-closed EEG was recorded to capture residual inhibition (RI). Immediately after TMNMT stimulation and EEG acquisition, patients rated tinnitus loudness and annoyance using a visual analog scale (VAS), and clinicians documented the depth and duration of RI based on patient reports. Residual inhibition (RI) was assessed immediately after the 10-min TMNMT stimulation. RI duration was obtained by asking patients to report the moment at which their tinnitus returned to its pre-stimulation baseline. Clinicians recorded the elapsed time from the end of stimulation to the subjective recovery of tinnitus perception. Both RI depth and RI duration were documented as part of the routine clinical evaluation. The effectiveness of TMNMT was further evaluated using a Likert scale ranging from −5 to 5, where −5 indicated complete suppression of tinnitus, 0 indicated no change, and 5 indicated a doubling of perceived loudness.

Classification of RI outcomes followed the criteria described by Hu et al. (2021). Patients were categorized as “RI positive” if they exhibited an averaged maximum RI depth of −4 or −5, corresponding to almost complete or complete tinnitus suppression. Those who did not meet this criterion—including individuals with minimal suppression (RI depth > − 1) or partial suppression (−4 < RI depth ≤ − 1)—were classified as “RI negative.”

### 2.4 Data collection procedure

*Pre-recording controls*: Participants were instructed to obtain adequate sleep and to abstain from smoking, caffeine, and strenuous physical or mental activity for at least 24 h prior to EEG recording.

*Setting and equipment*: EEG was recorded in a sound-attenuated and electromagnetically shielded room using an EGI GES 400 system (Electrical Geodesics, Inc., USA) with a 64-channel HydroCel sponge cap pre-soaked in potassium chloride (KCl) solution to improve electrode–skin conductance. Electrode impedances were maintained below 50 kΩ throughout data collection.

*Acquisition parameters*: Signals were acquired with Net Station Acquisition 5.4.3-R, using Cz as the online reference, at a sampling rate of 1 kHz, and with a 0.1–30 Hz band-pass. Electrodes were positioned according to the international 10–20 system.

Participant instructions during recording. During EEG acquisition, participants were asked to minimize facial, ocular, and neck movements. In eyes-open segments, a central white fixation cross was presented on the display, and participants were instructed to maintain fixation; eyes-closed segments were recorded while participants remained relaxed and still.

### 2.5 Power spectrum analysis

All electroencephalogram (EEG) data were processed and visualized using the EEGLAB v2021.0 toolbox in MATLAB R2023b (Delorme and Makeig, 2004). The data preprocessing pipeline was conducted as follows:

1. Filtering implementation: A 1 Hz high-pass filter, a 30 Hz low-pass filter, and a 50 Hz notch filter were applied to mitigate electrode drift, muscle noise, and power line interference, respectively;
2. The raw data were downsampled to a sampling rate of 250 Hz;
3. The continuous EEG signals were segmented into contiguous 2-s epochs;
4. Channel-level correction: Channels with poor signal quality were identified and then reconstructed via spherical interpolation, ensuring a consistent channel count across all participants;
5. Epoch-level rejection: Artifact-contaminated epochs associated with non-neuronal activities (e.g., swallowing, teeth clenching/gnashing, chewing, and excessive electromyographic (EMG) activity) were identified and discarded;
6. The original reference at the Cz electrode was re-referenced to the average potential of all electrodes (Hu et al., 2017);
7. Independent Component Analysis (ICA): ICA was performed to identify and remove independent components reflecting ocular activity (blinks/saccades), muscular activity, cardiac activity, head movements, channel noise, and other transient artifacts;
8. Based on predefined time markers, the processed EEG data were divided into three segments: Pre-stim, During-stim, and Post-stim;
9. A voltage threshold of ±100 μV was applied to screen epochs, with all epochs exceeding this threshold excluded from subsequent analyses.

This preprocessing pipeline effectively attenuated non-neuronal artifacts and enhanced data quality prior to subsequent analytical procedures.

Power spectral analysis were computed for each electrode and then averaged across all electrodes to obtain a global spectral estimate. The Spectral Power Ratio (SPR) was derived from this global average, as the goal of the present study was to assess overall oscillatory modulation rather than spatially localized effects. No electrode-specific or topographical analyses were performed. Power spectrum analysis was then performed using the Welch method to obtain the power spectrum within the frequency range of 1–30 Hz, where the vertical axis represents power (in μV^2^/Hz) and the horizontal axis represents frequency (in Hz). Four classic EEG frequency bands were defined as follows: delta (1–4 Hz), theta (4–8 Hz), alpha (8–13 Hz), and beta (13–30 Hz). The power of each frequency band was calculated by integrating the power spectrum curve over the corresponding frequency range (i.e., calculating the area under the curve within the specific band). The total power was determined as the sum of the powers of the four bands (equivalent to integrating the power spectrum over the entire 1–30 Hz range). Finally, the ratio of each band’s power to the total power was computed using the formula: (Power of target band / Total power) × 100%, expressed as a percentage. We can analyze changes in the activity intensity of a specific frequency band by means of the spectral power ratio (SPR).

### 2.6 Statistical analysis

Statistical analysis was conducted using SPSS 25.0 software. The quantitative data that conforms to a normal distribution is represented as x ± SD. Due to the small number of experimental samples, non-parametric tests were used in the SPR analysis (Mann and Whitney, 1947). Band-limited SPR (delta, theta, alpha, beta) across time (pre, during, post) was tested using the Friedman test, with post-hoc Wilcoxon signed-rank tests (pre vs. during, pre vs. post, during vs. post), and correction effects were applied to multiple comparisons of false discovery rate (FDR). Linear regression and correlation analysis were performed using GraphPad Prism 9.0 software, with statistical significance set at *p <* 0.05. Continuous variables were analyzed using *t*-test or Mann–Whitney U test, while categorical variables were analyzed using chi square test. One way analysis of variance was used to compare the clinical differences between RI positive and RI negative groups. Using binary logistic regression analysis to study the factors affecting RI positivity rate. RI outcomes were summarized as depth (ordinal scale) and duration (seconds).

## 3 Results

### 3.1 Demographics and clinical characteristics

Table 1 summarizes the baseline characteristics of the study cohort, including demographic information, tinnitus-related clinical measures (such as tinnitus duration, laterality, loudness, and matched frequency), audiometric thresholds, and other routine baseline assessments collected prior to TMNMT.

**Table 1:** The demographics and clinical characteristic of participants.

Variable	Value (*n =* 29)
Age (year, std)	51.59 ± 14.30
Gender (*n*, male/female)	29 (15/14)
Tinnitus duration (month, std)	24.03 ± 32.86
Pure-tone average (dB HL)	22.28 ± 7.56
Tinnitus lateralization (left/right/bilateral)	10/6/13
RI Result(positive/negative)	20/9
THI score (std)	38.97 ± 17.32
VAS loudness score (std)	5.38 ± 1.45
VAS annoyance score (std)	5.07 ± 1.36

This table presents descriptive information only; no statistical comparisons were performed.

### 3.2 Analysis of RI effect

#### 3.2.1 Analysis clinical characteristics between RI positive group and RI negative group

After 10 min of TMNMT, 20 patients (69%) showed positive results for residual inhibition (RI), while 9 patients (31%) showed negative results. According to the total THI score, the severity of tinnitus is classified into 5 levels: normal (0–16 points), mild (18–36 points), moderate (38–56 points), severe (58–76 points), and catastrophic (78–100 points) tinnitus (Takahashi et al., 2018; Reddy et al., 2019). The RI-negative group had an average THI score of (29.67 ± 13.34) points, and 33.3% of the patients fell within the moderate–to–catastrophic range (THI ≥ 38). In contrast, the RI-positive group showed a higher average THI score of (44.65 ± 15.77) points, and the proportion of moderate to catastrophic tinnitus cases reached 65.0%. Statistically significant differences were observed between the two groups (*p* = 0.020 and *p* = 0.048, respectively).

Regarding the duration of tinnitus, 44.4% of patients in the RI-negative group reported a duration exceeding 6 months, whereas this proportion was significantly higher in the RI-positive group at 90% (*p <* 0.05). There were no significant differences between the two groups in terms of average age [RI-negative: (49.85 ± 16.02) years; RI-positive: (55.00 ± 13.02) years, *p* = 0.406], gender distribution (*p* = 0.688), pure tone audiometry (PTA) results (*p* = 0.769), tinnitus lateralization (*p* = 1.0), tinnitus frequency (*p* = 0.201), tinnitus loudness (*p* = 0.568), VAS loudness score (*p* = 0.252), or VAS annoyance score (*p* = 0.335). These results are summarized in Table 2.

**Table 2:** Comparison of demographics and clinical characteristics between RI positive group and RI negative group.

Variable	RI negative (*n =* 9)	RI positive (*n =* 20)	*p*-value
Age (year, std)	49.85 ± 16.02	55.00 ± 13.02	0.406
Gender (*n*, %)			0.688
Male	4 (44.4)	12 (60.0)	
Female	5 (55.6)	8 (40.0)	
Tinnitus duration (*n*, %)			0.016*
<6 month	5 (55.6)	2 (10.0)	
≥6 month	4 (44.4)	18 (90.0)	
Pure Tone Average(dB HL)	21.92 ± 8.84	22.00 ± 7.15	0.769
Tinnitus lateralization (*n*, %)			1.0
Left	3 (33.3)	7 (35.0)	
Right	2 (22.2)	4 (20.0)	
Bilateral	4 (44.4)	9 (45.0)	
Tinnitus frequency			0.201
<4 k Hz	8 (88.9)	12 (60.0)	
≥4 k Hz	1 (11.1)	8 (40.0)	
Tinnitus loudness(dB SPL)	52.60 ± 11.86	55.22 ± 9.78	0.568
THI score (*n*, %)	29.67 ± 13.34	44.65 ± 15.77	
<36 points	6 (66.6)	7 (35.0)	0.020*
≥38 points	3 (33.3)	13 (65.0)	0.048*
VAS-L (point, std)	4.78 ± 0.83	5.25 ± 1.07	0.252
VAS-A (point, std)	4.89 ± 0.93	5.25 ± 0.91	0.335

*p < 0.05.

#### 3.2.2 Analysis of factors affecting RI positive rate

Select factors with positive results from the univariate analysis for the binary logistic regression analysis using the ‘inputs’, including tinnitus duration and the THI score. Although the overall logistic regression model did not reach statistical significance (*χ*^2^ = 2.537, df = 2, *p* = 0.281), the odds ratio for THI score was greater than 1, suggesting a possible trend toward higher RI positivity in patients with more severe tinnitus. This finding should be interpreted cautiously given the limited sample size.

#### 3.2.3 RI duration and intensity

After 10 min of TMNMT, 20 patients (69%) showed positive results for residual inhibition (RI), while 9 patients (31%) showed negative results. The duration of RI was also recorded, which was measured from the end of the sound stimuli until the level of tinnitus loudness returned to its initial level. The results for the entire group showed that TMNMT had a significant inhibition time (153 ± 210 s). The residual inhibition positive group showed longer inhibition times (222 ± 221 s). TMNMT had a strong inhibition intensity (−3.8 ± 1.7).

### 3.3 Behavioral results

**Figure 1:**
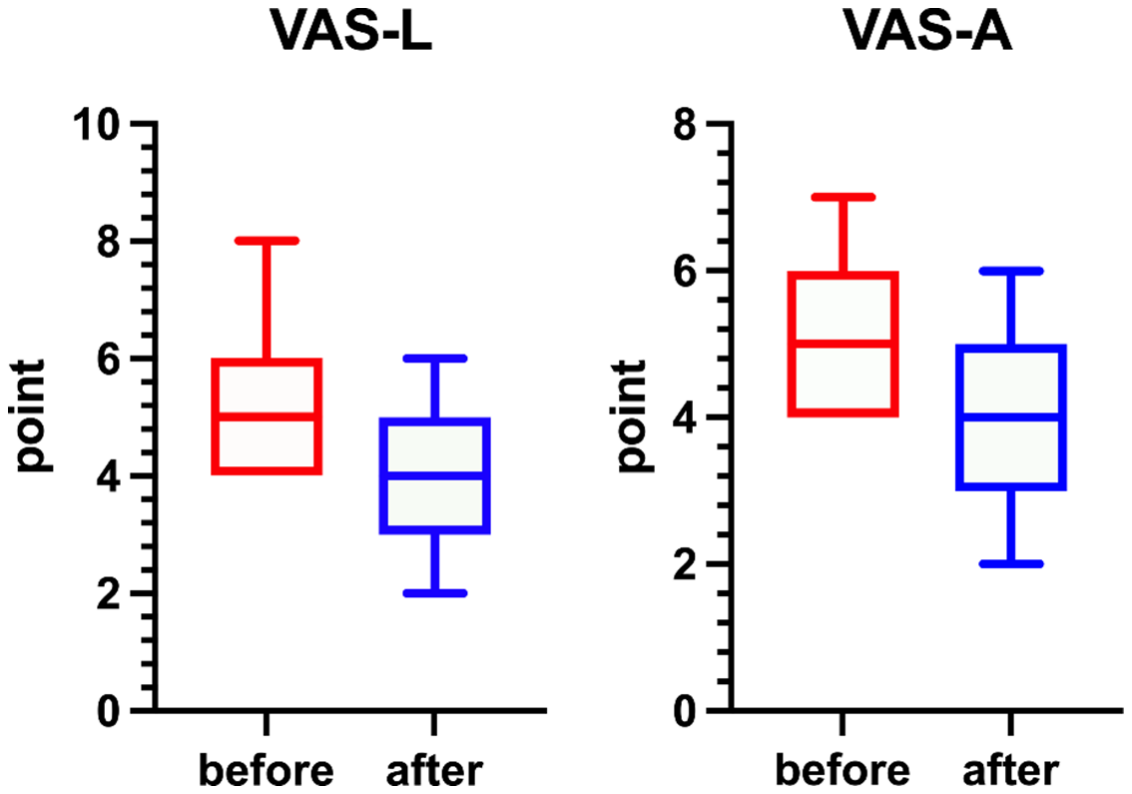
Changes in tinnitus loudness and annoyance scores before and after TMNMT intervention. Box plots illustrate individual variations in VAS-L (Visual Analog Scale for Loudness) and VAS-A (Visual Analog Scale for Annoyance) measured before (red) and after (blue) treatment. Both loudness and annoyance ratings significantly decreased after TMNMT, as confirmed by the Wilcoxon signed-rank test (*p <* 0.05), indicating a significant subjective improvement in tinnitus loudness and annoyance.

A paired-sample *t*-test of VAS scores before and after TMNMT stimulation showed VAS-L (t = 6.648, *p <* 0.001) was significantly reduced after treatment and VAS-A (t = 5.446, *p <* 0.001) (Figure 1).

### 3.4 Spectral power ratio analysis

**Figure 2:**
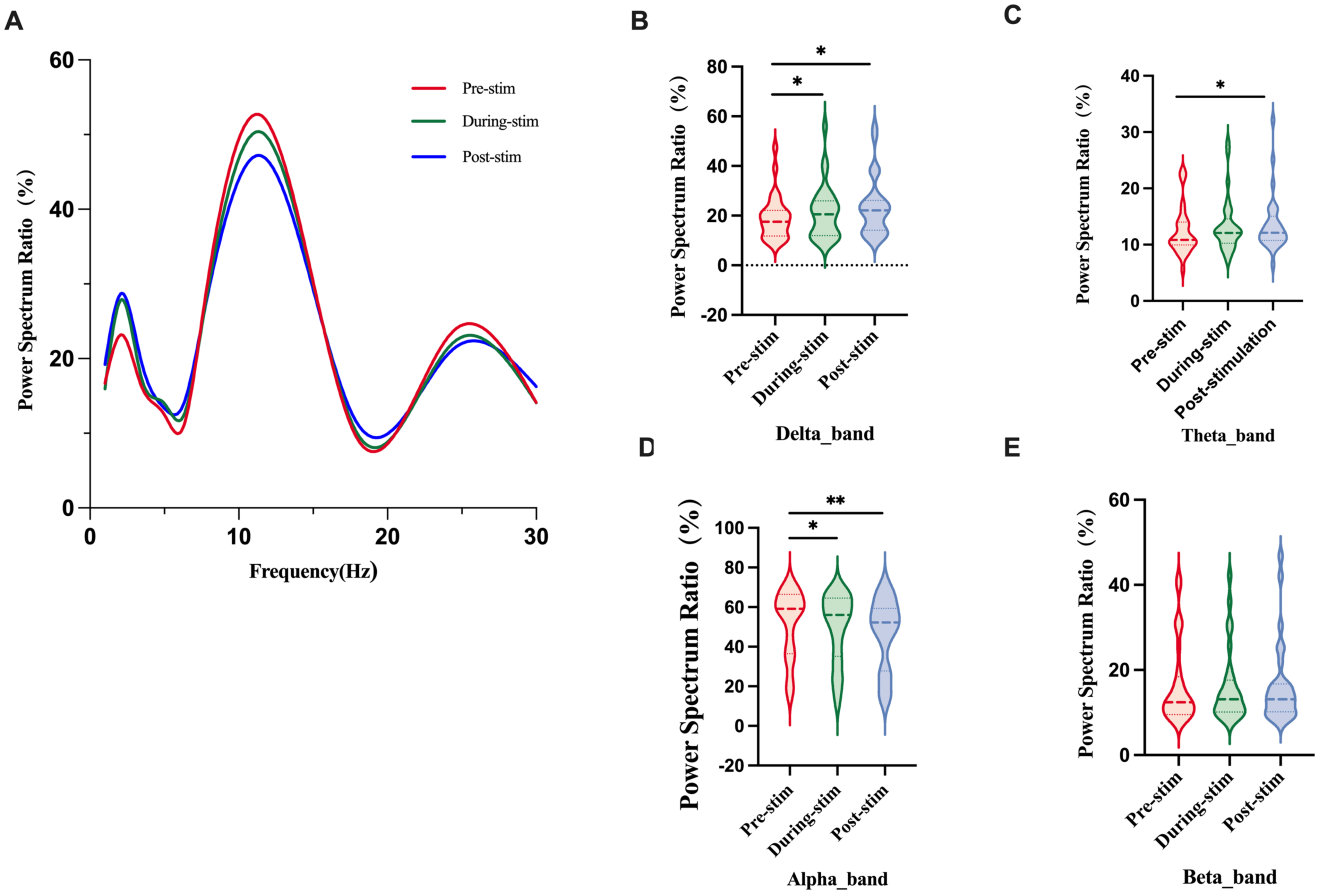
Power spectrum ratio across different frequency bands before, during, and post stimulation. **(A)** Grand-average power spectrum ratio (0–30 Hz) showing frequency-dependent changes across conditions. **(B–E)** Violin plots display group-level comparisons of the power spectrum ratio (%) among the three stimulation stages for each frequency band. Significant differences are indicated as *p < 0.05, p** < 0.01.

Due to the deviation of SPR value distribution from normality, Friedman test analysis was performed on the results of the subjects before, during, and after TMNMT stimulation (Figure 2). Figure 2A displays the grand-average power spectrum ratio (0–30 Hz) across all scalp electrodes for the pre-, during-, and post-stimulation conditions, providing an overview of the frequency-dependent SPR profile before examining band-specific differences. The results showed a significant increase in SPR in the *δ* and *θ* frequency bands, and a significant decrease in SPR in the *α* frequency band. Wilcoxon sign rank test was then used for pairwise comparison between groups. After FDR correction, the SPR of delta band (during-stim vs. pre-stim, *p* = 0.011; post-stim vs. pre-stim, p = 0.011; Figure 2B) and theta band (post-stim vs. pre-stim, *p* = 0.033; Figure 2C) were significantly increased, alpha band (during-stim vs. pre-stim, *p* = 0.029;post-stim vs. pre-stim, *p <* 0.001; Figure 2 D) was significantly decreased. No significant changes were observed in the beta band (during-stim vs. pre-stim, *p* = 0.606; post-stim vs. pre-stim, *p* = 0.854; Figure 2E). Besides, natural logarithmic transformation (ln transformation) was performed on the SPR value to improve its normality and used for two factor analysis of variance. The converted data was verified to follow a normal distribution (Shapiro Wilk test, *p* > 0.05). The frequency band (delta, theta, alpha, beta) was regarded as a within-subject factor and group (pre-stim, during-stim, post-stim) as a between-subject factor. From an analysis of the waveforms, Figure 2A showed the spectral power ratio (SPR) of whole brain. The results of SPR showed that the main effect of frequency band factor (*F* = 54.96; *p <* 0.0001) and frequency band Group interaction effect (*F* = 7.999; *p <* 0.0001) were significant. The main effect of group factor was not significant (*F* = 2.001; *p* = 0.1482) (Table 3).

**Table 3:** The spectral power ratio (SPR) of whole-brain certain frequency bands in subgroups.

SPR(%)Mean (Std)	Pre-stim (*n =* 29)	Dur*ing-stim* (*n =* 29)	Post-stim (*n =* 29)	z/p (pre -dur*ing*)	z/p (pre -post)	z/p (during-post)
Alpha	52.03 ± 16.94	49.4 ± 17.45	46.83 ± 18.04	−2.649/0.029*	−3.471/ <0.001	−1.849/0.117
Beta	16.42 ± 16.94	15.98 ± 8.74	16.33 ± 19.52	−0.768/0.606	−0.184/0.854	−0.681/0.606
Theta	12.37 ± 4.29	13.36 ± 4.87	13.63 ± 5.10	−2.174/0.066	−2.520/0.033*	−0.205/0.854
Delta	19.17 ± 8.80	21.2 ± 10.80	23.22 ± 11.60	−3.168/0.011*	−3.125/0.011*	−0.962/0.528

### 3.5 Correlation analysis

**Figure 3:**
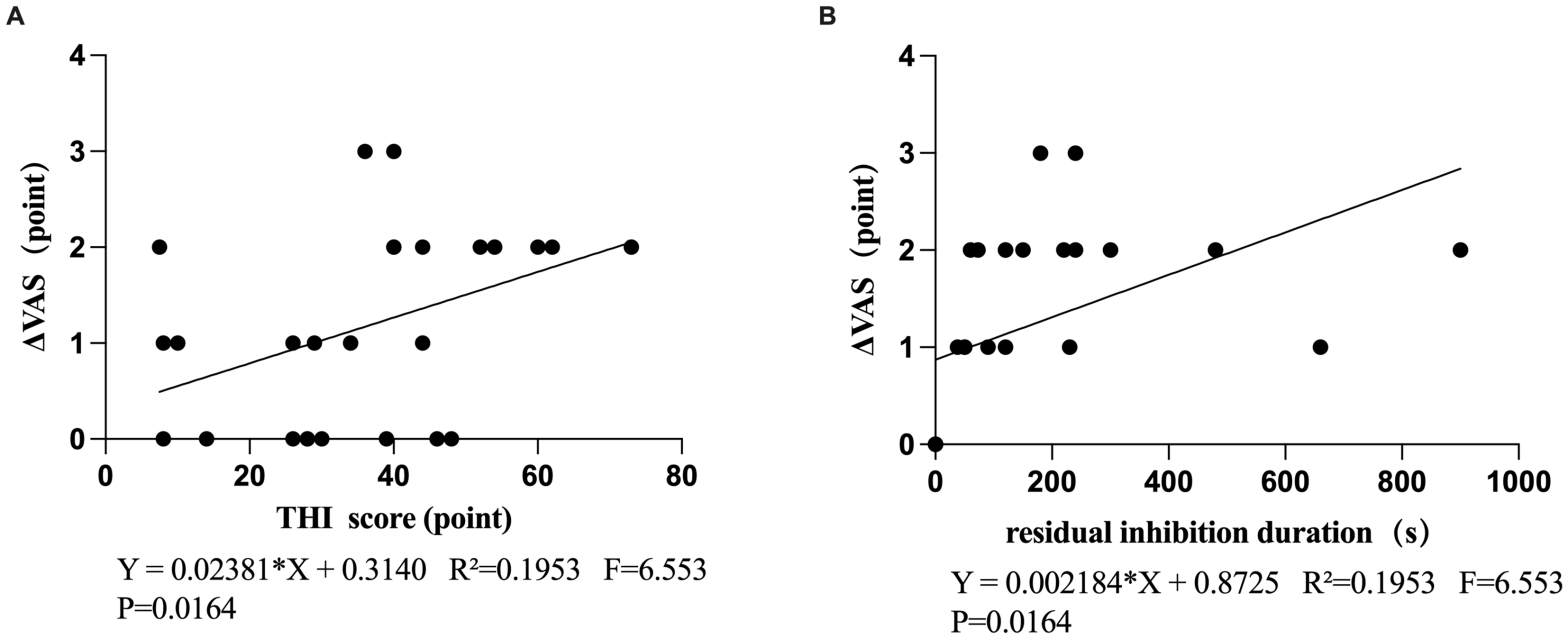
**(A)** Correlation between the THI score and ΔVAS **(B)** Correlation between the residual inhibition duration and ΔVAS.

This study examined the correlation between the changes in VAS scores (ΔVAS) before and after TMNMT stimulation and THI scores (before treatment), It was observed that there was a statistical significance in the increase of ΔVAS as THI score increased (Figure 3A). Further correlation analyses were conducted on the RI duration and ΔVAS, It was observed that there was a statistical significance in the increase of ΔVAS as the RI duration increased (Figure 3B).

## 4 Discussion

To our knowledge, this study is among the first to examine TMNMT-related residual inhibition using resting-state EEG spectral power ratio (SPR). Results showed the following:(1)10 min after TMNMT, SPR increased significantly in the delta and theta bands and decreased significantly in the alpha band; the beta band exhibited a downward trend that did not reach statistical significance.(2)Following TMNMT treatment, both VAS-I and VAS-A scores decreased significantly. This indicates a reduction in both the loudness of tinnitus and the subjective distress it causes, suggesting that TMNMT treatment exerts a positive effect on patients with tinnitus. (3)The higher the THI score (indicating more severe tinnitus), the greater the change in VAS scores induced by TMNMT. (4)There is no significant correlation between the Tinnitus Handicap Inventory (THI) score—an indicator of tinnitus severity—and the duration of residual inhibition following TMNMT treatment. Collectively, these data support the potential utility of TMNMT for tinnitus and extend current understanding of its underlying electrophysiology.

### 4.1 Why do we choose the spectral power ratio?

Previous studies have shown that tinnitus is often associated with abnormal activity in central brain regions, and resting state spectral features can serve as a biological indicator of subjective tinnitus (Moazami-Goudarzi et al., 2010). Most prior work has quantified spectral changes using power spectral density (PSD) (Zhu and Gong, 2023); or absolute band power (Vanneste and De Ridder, 2016; Kyong et al., 2024; Huang et al., 2021). Because PSD/absolute power is sensitive to inter-individual factors (e.g., skull conductivity, electrode impedance), absolute band powers vary widely across participants, which can obscure group-level patterns. The Relative Power, which indicates the ratio of the power of a frequency band to the total band power, could be used to reduce this problem (Ahn et al., 2013; Dohrmann et al., 2007; Güntensperger et al., 2019; Jensen et al., 2023).

Although the SPR definition varies across studies (e.g., (*α* + *β*)/(*δ* + *θ*), α/δ, θ/β), these ratio-based metrics share the same rationale of normalizing band-limited activity to reduce inter-individual variability and to emphasize relative spectral redistribution across bands. Prior electrophysiological work has demonstrated that the balance between slow-wave activity (δ, θ) and faster rhythms (α, β) reflects large-scale changes in cortical excitability and network dynamics (Steriade, 2006; Nunez, 1981; Benoit et al., 2000; Uchida et al., 1991). In tinnitus research, characteristic patterns such as increased δ–θ activity accompanied by reduced α power have been repeatedly observed (Moazami-Goudarzi et al., 2010; Weisz et al., 2005), suggesting that changes in the *relative* contribution of slow versus fast oscillations may better capture the underlying pathophysiology than absolute power alone. Spectral ratio metrics have also been shown to provide sensitive indicators of physiological state transitions in cognitive and sensory domains (Ahn et al., 2013; Puma et al., 2018; Gevins, 1997). Accordingly, the SPR formulation adopted here serves as a normalized index representing the proportion of slow-wave activity within the total spectral power, thereby highlighting global shifts in inhibitory–excitatory balance that may occur during and after TMNMT.

In the present study, spectral power ratio (SPR) was selected as the primary EEG metric to characterize oscillatory changes associated with TMNMT-induced residual inhibition. Unlike conventional PSD-based band power, which reflects absolute or regional power changes, SPR provides a normalized measure of relative spectral redistribution across frequency bands (Kopčanová et al., 2024; Zawiślak-Fornagiel et al., 2023). This ratio-based approach is particularly suitable for heterogeneous clinical populations, such as tinnitus patients, in whom inter-individual variability in absolute EEG power can be substantial. By normalizing each frequency band to the total broadband power, SPR emphasizes cross-band modulation and global excitatory–inhibitory balance rather than absolute power magnitude. Accordingly, the present EEG findings should be interpreted as reflecting relative changes in spectral composition rather than absolute increases or decreases in specific frequency bands.

Within this framework, we observed a frequency-specific pattern characterized by increased delta and theta SPR accompanied by reduced alpha SPR during and after TMNMT exposure. This pattern is broadly consistent with models of tinnitus-related thalamocortical dysrhythmia, in which alterations in low-frequency and alpha-band activity are thought to reflect changes in inhibitory–excitatory balance within auditory and non-auditory networks. Importantly, the observed modulation occurred on a short timescale and was temporally aligned with behavioral residual inhibition, suggesting that TMNMT may transiently reshape large-scale oscillatory dynamics rather than inducing sustained changes in absolute band power.

For completeness, we additionally present a conventional PSD-based band-power analysis in the Supplementary Material. As shown in the supplemental figure, absolute band-power measures yield a partially different pattern compared with the SPR-based results. This divergence does not indicate inconsistency, but rather reflects the fact that PSD and SPR capture complementary but non-identical aspects of neural oscillatory activity. Whereas PSD emphasizes absolute power changes within specific frequency bands, SPR highlights relative spectral redistribution across bands. Accordingly, the supplemental PSD analysis is intended to illustrate the metric-dependent nature of spectral interpretation, while all primary conclusions of the present study are based on SPR.

### 4.2 Changes in SPR

This study found that, relative to pre-stimulation, *δ*-band SPR increased significantly both during TMNMT and in the 10-min post-stimulation period. As part of the slow-wave range, δ oscillations characteristically emerge in cortical regions lacking thalamocortical afferent input (Steriade, 2006). Low-threshold spike (LTS) bursts in thalamic nuclei—triggered when neurons become hyperpolarized because of deafferentation or excessive inhibition—can amplify cortical slow waves (Weisz et al., 2007). Fundamentally, *δ* activity reflects the oscillatory dynamics of deafferented or deprived neural networks (Llinás et al., 2005), and fluctuations in oscillatory EEG power generally index changes in population-level neuronal synchrony (Nunez, 1981; Bickford et al., 1979). In tinnitus, increases in *δ* and *θ* power accompanied by decreases in *α* power are associated with the presence and severity of the percept; δ enhancement typically co-occurs with α reduction—a pattern consistent with the present findings (Uchida et al., 1991; Benoit et al., 2000; Weisz et al., 2005). In studies evaluating electrophysiological changes associated with improvement in tinnitus perception via auditory coordinated reset (CR) stimulation (Tass et al., 2012), pretreatment pathological delta- and gamma-band activity normalized only in treatment responders (Adamchic et al., 2014), suggesting that thalamocortical dysrhythmia (TCD) may be causally linked to tinnitus. Consistent with this, a TMNMT intervention study reported significant increases in relative *δ*- and θ-band power in the treatment group, suggesting central cortical remodeling that may alter thalamocortical connectivity and, in turn, tinnitus-related neural networks (Zhu and Gong, 2023)^.^ δ-band alterations are further linked to functional connectivity within tinnitus-related networks, including interactions between the auditory cortex and limbic structures (Adamchic et al., 2014; Vanneste and De Ridder, 2011). Viewed through the lens of thalamocortical dysrhythmia, TMNMT may suppress aberrant fast activity (e.g., ***γ*** range) and shift thalamocortical rhythms from pathological high-frequency dominance toward physiological slow-wave dominance, thereby increasing the proportion of δ-band power.

In this study, we observed a significant increase in theta-band SPR after 10 min of TMNMT stimulation. This pattern aligns with Gong et al. (Zhu and Gong, 2023)^,^ who likewise reported a significant increase in theta-band PSD in the TMNMT group. Note that SPR (spectral power ratio) indexes relative band power—i.e., theta power normalized to total or reference-band power—whereas PSD reflects absolute power; despite these different scales, both metrics are sensitive to band-limited activity, so an increase in theta SPR is expected to co-vary with increases in theta PSD when the normalization is stable. Patho-physiologically, tinnitus has been attributed to bottom-up deafferentation and/or deficits in top-down noise cancellation. Both mechanisms are thought to alter thalamocortical signaling and give rise to thalamocortical dysrhythmia (TCD). Under deafferentation, TCD is characterized by a resting-state slowing from alpha to theta activity together with enhanced surrounding gamma activity, yielding persistent theta–gamma cross-frequency coupling (De Ridder et al., 2015; Weisz et al., 2007; De Ridder et al., 2011). This framework has been corroborated by EEG, MEG, and intracranial recordings (Weisz et al., 2007; Llinás et al., 2005; De Ridder et al., 2011; van der Loo et al., 2009; Llinás et al., 1999). Information streams linked to tinnitus, pain, movement, and emotion conveyed by high-frequency rhythms (beta/gamma) can be nested within theta via cross-frequency coupling (De Ridder and Vanneste, 2014). Against this background, the post-TMNMT elevation in theta power may index a transient reorganization of neuronal synchrony that could disturb the persistent theta–gamma coupling underlying the tinnitus percept (Weisz et al., 2007). It may also reflect a temporary rebalancing between excitatory and inhibitory neuronal ensembles, in line with observations during residual inhibition (Kahlbrock and Weisz, 2008). Additionally, theta oscillations support memory retrieval and large-scale network synchronization (Eggermont and Tass, 2015); thus, the enhanced theta activity observed here may suggest activation of Para hippocampal–auditory cortical connections involved in retrieving auditory information that is missing due to deafferentation (De Ridder et al., 2015).

In our study, alpha-band SPR decreased significantly both during TMNMT and after the stimulation period. This pattern aligns with Gong et al. (Zhu and Gong, 2023), who likewise reported a significant reduction in alpha-band PSD in the TMNMT group. Functionally, alpha oscillations are widely interpreted as an index of cortical inhibition: elevations in alpha power are observed under fatigue and are thought to downregulate processing of external information to limit cognitive load (Käthner et al., 2014; Puma et al., 2018; Gevins, 1997; Lou et al., 2025; Lorist et al., 2000). Conversely, lower alpha power is associated with active neural processing and better perceptual performance (Lorist et al., 2000; Gevins, 1997)^,^ whereas higher alpha indicates active suppression in task-irrelevant regions (Lorist et al., 2000; Gevins, 1997). In tinnitus, increased alpha1、alpha2 activity has been linked to greater tinnitus-related distress (Vanneste et al., 2010). Against this background, the TMNMT-induced reduction in alpha power observed here may index a normalization of cortical dynamics—i.e., reduced need for tonic inhibitory control—allowing more efficient sensory–attentional processing and, potentially, less attentional capture and distress associated with the tinnitus percept.

In this study, the *β*-band spectral power ratio (SPR) showed a downward trend 10 min after TMNMT stimulation, although the change did not reach statistical significance. Rather than claiming full consistency with prior work, we note that Zhu and Gong (2023) reported a non-significant reduction in β-band power spectral density (PSD) 5 min after TMNMT; despite differences in time points (5 vs. 10 min) and metrics (PSD vs. SPR), the effects were directionally similar. Converging evidence links anxiety traits, frontoparietal β activity, and hyperacusis (Inguscio et al., 2024). In the resting state, patients with tinnitus have higher power in the β band than healthy controls, suggesting that this may be related to fatigue and emotional effects produced by tinnitus (Vanneste et al., 2010; Meyer et al., 2014; Meyer et al., 2017). And greater distress has been associated with increased β activity (Jokić-begić and Begić, 2003; Begić et al., 2001). Against this background, the observed post-TMNMT reduction in β-band SPR—albeit nonsignificant—may indicate a transient modulation of neural processes related to distress and anxiety, suggesting that TMNMT could help alleviate the perceived burden of tinnitus. Larger samples and longer intervention windows are needed to confirm the effect size and clinical relevance.

### 4.3 RI effect

Subjective tinnitus is a common clinical condition frequently accompanied by anxiety, depression, sleep disturbance, and autonomic dysfunction; in severe cases, maladaptation may lead to self-harm or suicidal ideation. Sound-based therapy can alleviate the adverse experience of tinnitus and, to some extent, reduce perceived loudness. In this retrospective study, we observed that a subset of patients exhibited residual inhibition (RI) after tailor-made notched music training (TMNMT), suggesting potential therapeutic benefit. Our aim was to summarize RI outcomes among patients of this project and to conduct a preliminary analysis of factors associated with RI in response to TMNMT, to inform future clinical application.

We included 29 patients in total; 20 patients (69%) showed a positive RI response, whereas 9 (31%) did not. Univariate analyses indicated that the RI-positive group had higher baseline Tinnitus Handicap Inventory (THI) scores and a greater proportion with symptom duration exceeding six months. In binary logistic regression, baseline THI was not a statistically significant predictor; however, the odds ratio for THI was greater than 1, suggesting a trend toward a higher RI-positive rate among patients with moderate–to–severe tinnitus compared with those with mild tinnitus. Prior studies have similarly reported that greater baseline tinnitus severity predicts better response to sound-based interventions. For example, Theodoroff et al. (2014) found that patients who perceived their tinnitus as more severe had approximately 3.8-fold higher odds of treatment effectiveness relative to those with milder symptoms, and Cai et al. (2017) reported that each 1-point increase in baseline THI was associated with a 4% increase in the probability of therapeutic benefit with masking therapy. To mitigate small-sample effects in our cohort, we categorized patients by THI into mild versus moderate–to–severe (often corresponding to compensated vs. decompensated tinnitus, respectively). We observed a higher treatment response in the decompensated (moderate–to–severe) group than in the compensated (mild) group. Collectively, these findings are consistent with the notion that greater tinnitus severity may be associated with better response to TMNMT therapy.

Having shown that TMNMT can elicit RI in a proportion of patients and having preliminarily explored potential correlates of RI, our results should be interpreted with caution given the limited sample size. Future work with larger cohorts and more comprehensive covariate collection will be valuable to derive more reliable estimates and to delineate patient characteristics most likely to benefit from TMNMT, thereby providing practical guidance for clinicians.

## 5 Limitations and future work

This study has several limitations. First, as a retrospective analysis based on routine clinical data, no control condition (e.g., unmodified music or silence) was available for comparison, and causal conclusions regarding TMNMT effects should therefore be drawn with caution. Second, the modest sample size and heterogeneity of tinnitus characteristics may limit generalizability. Third, all EEG analyses were performed at the scalp level, which constrains spatial resolution and prevents anatomical interpretation of the observed oscillatory changes.

The present work focused on global spectral power ratio (SPR) as a normalized index of frequency-specific redistribution rather than on regional or source-resolved power measures. Accordingly, scalp-level power spectral density (PSD) topographies are provided only as supplementary descriptive material and should not be interpreted as evidence of spatially localized neural generators. Future prospective studies with larger cohorts, appropriate control conditions, and advanced analytical approaches—such as source localization, functional connectivity, and EEG microstate analysis—will be essential to further elucidate the neural mechanisms underlying TMNMT-related modulation and residual inhibition.

## 6 Conclusion

In this retrospective analysis, tinnitus relief and residual inhibition were observed in a proportion of patients during the routine TMNMT evaluation protocol. These perceptual changes were accompanied by frequency-specific variations in spectral power ratios across pre-, during-, and post-stimulation EEG recordings. Exploratory analyses further suggested that individuals with higher baseline tinnitus severity were more likely to exhibit within-session improvements and positive RI responses. While causal interpretations cannot be drawn, these findings characterize neural activity patterns associated with short-term tinnitus suppression and provide preliminary observational evidence supporting the potential clinical relevance of TMNMT. Further confirmation in larger cohorts with controlled study designs is warranted.

## Data Availability

The original contributions presented in the study are included in the article/supplementary material, further inquiries can be directed to the corresponding authors.
